# Eldecalcitol, an Active Vitamin D_3_ Derivative, Prevents Trabecular Bone Loss and Bone Fragility in Type I Diabetic Model Rats

**DOI:** 10.1007/s00223-017-0298-8

**Published:** 2017-06-17

**Authors:** Satoshi Takeda, Mitsuru Saito, Sadaoki Sakai, Kenji Yogo, Keishi Marumo, Koichi Endo

**Affiliations:** 1 Product Research Department, Fuji Gotemba Research Laboratories, Chugai Pharmaceutical Co., Ltd, 1-135 Komakado, Gotemba, Shizuoka, 412-8513 Japan; 2 Department of Orthopedic Surgery, Jikei University School of Medicine, 3-25-8 Nishishinbashi, Minato-ku, Tokyo, 105-8461 Japan; 3 Medical Science Department, Chugai Pharmaceutical Co., Ltd, 2-1-1 Nihombashi Muromachi, Chuo-ku, Tokyo, 103-8324 Japan

**Keywords:** Eldecalcitol, Diabetic osteoporosis, Streptozotocin, Bone quality

## Abstract

Diabetes mellitus is known to adversely affect the bones and be associated with increased fracture risk. We examined whether eldecalcitol (ELD), an active vitamin D_3_ derivative, could inhibit the diabetic bone loss in streptozotocin-induced type I diabetic rats. ELD (10, 20, or 40 ng/kg), alfacalcidol (ALF; 25, 50, or 100 ng/kg), or vehicle was administered 5 times per week for 12 weeks from 1 week after diabetes induction. Normal control rats received the vehicle. Bone turnover markers, bone mineral density (BMD), and biomechanical strength of the lumbar spine and femur were measured, and bone histomorphometry was performed. Content of advanced glycation end products (AGEs) in the femoral shaft was also determined. In diabetic rats, serum osteocalcin (OC) concentration was lower and urinary excretion of deoxypyridinoline (DPD) tended to be higher than in normal rats. Areal BMD and maximum load of the lumbar vertebrae and femoral shaft were lower in diabetic rats than in normal rats. All doses of ELD and the highest dose of ALF reduced urinary DPD excretion, but had no effect on serum OC. The 20 and 40 ng/kg doses of ELD prevented decreases in BMD and the highest dose of ELD prevented the reduction in maximum load of the lumbar vertebrae, while ALF did not change these parameters. ELD and ALF did not affect areal BMD or biomechanical strength of the femoral shaft. In diabetic rats, bone volume and trabecular thickness in the trabecular bone of the lumbar vertebrae decreased and trabecular separation increased compared to normal rats. ELD and ALF prevented diabetes-induced deterioration of trabecular microstructure. AGE content in the femoral cortical bone increased in the diabetic rats, and ELD and ALF did not change AGE content compared to the diabetic rats. These results indicated that ELD suppressed bone resorption and prevented trabecular bone loss and deterioration of trabecular microstructure, resulting in prevention of reduction in biomechanical strength in type I diabetic rats.

## Introduction

It is known that both type I and type II diabetes mellitus (DM) are associated with an increased risk of fractures [1–3]. Although bone mineral density (BMD) is a major determinant of bone strength and fracture risk, bone quality is also an important factor contributing to bone strength and resistance to fracture [4–6]. Reduction in bone quality is suggested to play a prominent role in osteoporosis in DM patients. For example, fracture risk in type II DM patients has been shown to be higher than in non-DM subjects, despite BMD in type II DM patients being no less or even higher than in non-DM subjects  [7–9]. Increased fracture risk in type I DM patients, who frequently have low BMD, is also not fully explained by BMD [7]. Deterioration of bone microstructure and geometry and alteration of enzymatic and non-enzymatic collagen crosslinks in bone play important roles in the reduction of bone quality in DM [10–13].

Eldecalcitol (ELD), 2β-(3-hydroxypropyloxy)-1α,25-dihydroxyvitamin D_3_, is an active vitamin D_3_ analogue that is widely used in Japan for the treatment of osteoporosis. In a randomized double-blinded trial, ELD was demonstrated to be superior to alfacalcidol (ALF; 1α-hydroxyvitamin D_3_, a prodrug of active vitamin D_3_) in terms of increases in BMD and reduction in fractures [14]. In a clinical study, ELD was found to have an advantage over ALF in terms of improvement of bone geometry and biomechanical parameters, such as cross-sectional area, cortical thickness, cross-sectional moment of inertia, and section modulus, of the femoral neck and shaft [15].

ELD was shown to suppress bone resorption and increase BMD to a greater extent than ALF in ovariectomized (OVX) rats [16]. ELD increases bone minimodeling, a type of bone formation that is independent of bone resorption, in OVX rats and OVX monkeys [17–20]. In minimodeling, “bud-shaped” bone is deposited on quiescent surfaces and is therefore seen with a smooth cement line in histological observations [18]. It is also reported that ELD improved bone quality in OVX monkeys by increasing mineralization, suppressing accumulation of microdamage, increasing enzymatic collagen crosslinks, and decreasing non-enzymatic advanced glycation end product (AGE) crosslinks [20].

Although the beneficial effects of ELD on bone metabolism have been reported in OVX animals, there are no reports concerning the effect of ELD on bone in DM model animals. The streptozotocin (STZ)-induced DM rat is one of the most commonly used type I DM animal models and is used for the investigation of the influence of drugs on diabetic osteopenia [21–24]. In this study, we examined the effect of ELD on BMD, bone biomechanical strength, and bone quality in STZ-induced DM rats and compared the results with those of ALF. Bone quality was evaluated by analysis of bone histomorphometry and collagen crosslinks.

## Materials and Methods

### Reagents

ELD and ALF, both synthesized by Chugai Pharmaceutical Co., Ltd., were dissolved in medium-chain triglyceride and diluted to the given concentrations.

### Experimental Design

Ten-week-old male Sprague–Dawley rats (Crl: CD(SD)) were purchased from Charles River Laboratories Japan Inc. (Yokohama, Japan) and were acclimatized for 2 weeks under standard laboratory conditions at 20–26 °C, 30–70% humidity, and a 12-h:12-h light/dark cycle. All animals had free access to tap water and a commercial standard rodent chow (CE-2; CLEA Japan, Inc., Tokyo, Japan). DM was induced by a single intravenous injection of STZ (35 mg/kg; Wako Pure Chemical Industries, Osaka, Japan) dissolved in saline containing 1 mM citrate buffer, pH 4.5. Normal control animals received the STZ diluent. One week after STZ injection, blood glucose was measured using a glucometer (Accu-Chek Aviva; Roche Diagnostics K. K, Tokyo, Japan), and rats with blood glucose concentrations higher than 250 mg/dL were regarded as having DM. DM rats were divided into 7 groups of 12 animals, and orally administered vehicle, ELD (10, 20, or 40 ng/kg), or ALF (25, 50, or 100 ng/kg) 5 times per week for 12 weeks. In the normal control group, rats received the vehicle (1 mL/kg). Tetracycline (20 mg/kg) and calcein (6 mg/kg) were injected subcutaneously for bone labeling at 8 or 9 days and 2 or 3 days, respectively, prior to necropsy.

Blood and urine were collected every 4 weeks. Blood was collected from the jugular vein under isoflurane anesthesia and centrifuged to obtain serum. Urine was collected over a 24-h period using individual metabolic cages. Serum and urine were stored at −40 °C until biochemical analysis.

After 12 weeks of treatment, animals were euthanized by exsanguination from the abdominal aorta under isoflurane anesthesia. The lumbar spine and bilateral femurs were excised. The second through fourth lumbar vertebrae (L2–L4) and the right femur were retained in 70% ethanol. The fifth lumbar vertebra (L5) and the left femur were wrapped in saline-soaked gauze and stored at −40 °C prior to biomechanical testing. This study was performed according to the experimental protocol approved by the Institutional Animal Care and Use Committee at Chugai Pharmaceutical Co., Ltd.

### Biochemical Analysis

Glycosylated hemoglobin A1c (HbA1c), serum calcium (Ca), urinary Ca, and urinary creatinine (Cr) were measured with an autoanalyzer (TBA-120FR; Toshiba Medical Systems Co., Tochigi, Japan). Urinary deoxypyridinoline (DPD) was measured as a bone resorption marker using an Osteolinks-DPD kit (DS Pharma Biomedical, Osaka, Japan) and the data were corrected for urinary Cr concentration. Serum osteocalcin (OC) was measured as a bone formation marker using an Osteocalcin Rat ELISA System (GE Healthcare Japan Co., Tokyo, Japan).

### BMD Analysis

The areal BMD of the L2–L4 vertebrae was measured by dual-energy X-ray absorptiometry using a DCS-600EX bone densitometer (Aloka Co., Ltd., Tokyo, Japan). The areal BMD of the right femur was measured with the DCS-600EX and the scanning started at the most proximal area, including the femoral head, and ended at the most distal area, including the femoral condyles. During data analysis, the femur was divided into 10 equal segments along its major axis. The mean value of BMD was calculated in 3 parts, proximal (the 3 proximal segments of the scanned area), middle (the 4 middle segments of the scanned area), and distal (the 3 distal segments of the scanned area).

### Bone Histomorphometry

Bone histomorphometry was performed on the L3 vertebra and femoral diaphysis. Specimens were fixed in 70% ethanol and stained with Villanueva bone stain. After dehydration, the specimens were infiltrated with and embedded in methyl methacrylate. Five-micrometer-thick sagittal sections of the L3 vertebral body were prepared to evaluate trabecular bone. For the femoral diaphysis, 10- to 15-μm-thick cross-cut ground sections were obtained for the evaluation of cortical bone. Measurements of static and dynamic parameters were collected with a histomorphometric system (System Supply Co. Ltd., Nagano, Japan) linked to a microscope equipped with bright and epifluorescence illumination. The following variables were measured: bone volume (BV/TV, %), trabecular thickness (Tb.Th, μm), trabecular number (Tb.N,/mm), trabecular separation (Tb.Sp, μm), osteoclast surface (Oc.S/BS,  %), eroded surface (ES/BS,  %), osteoblast surface (Ob.S/BS,  %), osteoid surface (OS/BS,  %), mineralizing surface (MS/BS,  %), mineral apposition rate (MAR, μm/day), bone formation rate (BFR/BS, mm^3^/mm^2^/year), tissue area (T.Ar, mm^2^), marrow area (Ma.Ar, mm^2^), cortical area (Ct.Ar, mm^2^), and cortical width (Ct.Wi, mm). Nomenclature and units used in this study follow the report of the American Society for Bone and Mineral Research Histomorphometry Nomenclature Committee [25]. Minimodeling-based bone formation, a type of focal bone formation, was measured as previously reported [18, 19]. Briefly, a minimodeling site was defined as one with a smooth cement line and no interruption of the surrounding collagen fibers. The following parameters were measured: number of minimodeling sites per bone surface (N.MI/BS,/mm), minimodeling bone volume per total bone volume (MI.BV/BV, %), and minimodeling bone surface per total bone surface (MI.BS/BS,  %).

### Biomechanical Analysis

Biomechanical testing was performed using a mechanical testing machine (TK-252C; Muromachi Kikai Co., Ltd., Tokyo, Japan). For the femur 3-point bending test, the upper loading device was aligned at the expected breaking point of the femoral shaft on the anterior side. The span between the two lower supports was set at 12 mm. The load was applied at a rate of 20 mm/min until failure occurred. Prior to the compression test of the L5 vertebral body, the vertebral arch and end plates were removed to obtain a specimen with planoparallel ends. The loading rate of the compression test was set at 2.5 mm/min. Maximum load and work to failure were determined from the load–displacement curve, and ultimate stress and toughness were calculated [26]. Stiffness was determined from the slope of the linear elastic region of the load–displacement curve, and Young’s modulus was calculated. Prior to the 3-point bending test of the femoral shaft, cross-sectional moment of inertia and BMD were measured at the expected breaking point by peripheral quantitative computed tomography (pQCT; XCT Research M; Stratec Medizintechnik, Pforzheim, Germany) using CortMode 1. BMD at the mid-section of the L5 vertebral body was quantified by pQCT using ContMode 2 and PeelMode 20 (trabecular area, 45%).

### Collagen Crosslink Analysis

The analysis of collagen crosslinks in bone was performed according to the procedure reported previously [27]. Briefly, bone powder of the femoral shaft after biomechanical testing was prepared and demineralized twice with 0.5 M EDTA in 50 mM Tris buffer (pH 7.4) for 96 h at 4 °C. The demineralized bone residues were then suspended in 0.15 M potassium phosphate buffer (pH 7.6) and reduced at 37 °C with NaBH_4_. The reduced specimens were hydrolyzed in 6N HCl at 110 °C for 24 h. Hydrolysates were analyzed for the content of crosslinks and hydroxyproline on a Shimadzu LC9 HPLC fitted with a cation exchange column (0.9 × 10 cm, Aa pack-Na; JASCO, Ltd., Tokyo, Japan) linked to an inline fluorescence flow monitor (RF10AXL; Shimadzu, Shizuoka, Japan). The total content of AGEs was measured by the method of Saito et al. [20]. Briefly, AGE content was determined using a fluorescence reader at 370 nm excitation and 440 nm emission (JASCO FP6200; JASCO) and normalized to a quinine sulfate standard.

### Statistical Analysis

Data are expressed as the mean ± SEM. Differences between groups were analyzed with one-way analysis of variance (ANOVA). Statistical differences between the vehicle group and ELD-treated or ALF-treated groups were evaluated with Dunnett’s multiple comparison test. Statistical comparisons between the normal control group and the vehicle group were performed by unpaired *t* test. For all tests, *P* < 0.05 was considered statistically significant. Statistical analysis was carried out using the Statistical Analysis System software package or JMP (SAS Institute Inc., Cary, NC, USA).

## Results

### Body Weight and Physiological Parameters

The body weight in the vehicle group was significantly lower than that in the normal group (Table 1). Both blood glucose and HbA1c in the vehicle group were significantly higher than in the normal group. ELD and ALF had no significant effects compared to the vehicle group on body weight, blood glucose, or HbA1c (Table 1). Serum Ca in the vehicle group was similar but urinary Ca/Cr was significantly higher compared to the normal group. Serum Ca was not changed by ELD but was increased by 50 and 100 ng/kg of ALF treatment. In the 20 and 40 ng/kg ELD-treated and 100 ng/kg ALF-treated groups, urinary Ca/Cr was significantly higher than in the vehicle group (Table 1).

**Table 1:** Body weight and physiological parameters after 12 weeks of treatment

	Normal	STZ						
		Vehicle	ELD (ng/kg)			ALF (ng/kg)		
			10	20	40	25	50	100
Body weight (g)	601.8 ± 14.8	405.7 ± 9.2^a^	411.6 ± 10.2	399.9 ± 9.1	404.2 ± 14.8	420.6 ± 13.8	416.2 ± 14.4	400.4 ± 14.2
Blood glucose (mg/dL)	128 ± 1	421 ± 9^a^	394 ± 10	393 ± 13	395 ± 19	386 ± 17	438 ± 15	404 ± 14
HbA1c (%)	1.56 ± 0.02	4.60 ± 0.20^a^	4.41 ± 0.18	4.33 ± 0.25	4.31 ± 0.15	4.46 ± 0.20	4.65 ± 0.19	4.23 ± 0.24
Serum Ca (mg/dL)	10.0 ± 0.1	9.8 ± 0.0	9.9 ± 0.1	10.1 ± 0.2	10.3 ± 0.2	9.9 ± 0.1	10.1 ± 0.1^b^	10.4 ± 0.1^b^
Urinary Ca/Cr	0.02 ± 0.00	0.79 ± 0.14^a^	1.21 ± 0.19	2.13 ± 0.32^b^	2.37 ± 0.22^b^	1.19 ± 0.14	1.31 ± 0.24	2.44 ± 0.31^b^

Data are presented as mean ± SEM (*n* = 9–12)

*STZ* streptozotocin, *ELD* eldecalcitol, *ALF* alfacalcidol, *HbA1c* glycosylated hemoglobin A1c, *Ca* calcium, *Cr* creatinine

^a^ *P* < 0.05 versus normal rats by unpaired *t* test

^b^ *P* < 0.05 versus vehicle-treated rats by Dunnett’s multiple comparison test

### BMD

**Fig. 1:**
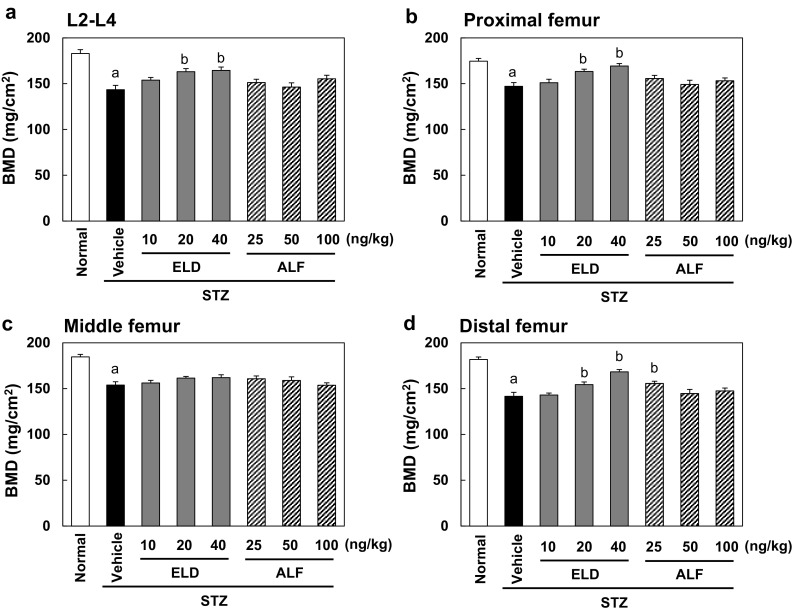
Areal BMD of L2–L4 vertebrae and femur after 12 weeks of treatment. Diabetes was induced in rats by streptozotocin (STZ) injection. Rats were treated with eldecalcitol (ELD) or alfacalcidol (ALF) for 12 weeks from 1 week after diabetes induction. **a** L2–L4 vertebrae, **b** proximal femur, **c** middle femur, and **d** distal femur. Normal, non-DM rats; Vehicle, DM rats treated with vehicle. Data are presented as mean ± SEM (*n* = 9–12). ^a^ *P* < 0.05 versus normal rats by unpaired *t* test, ^b^ *P* < 0.05 versus vehicle-treated rats by Dunnett’s multiple comparison test

The areal BMD of the L2–L4 vertebrae and the femur in the vehicle group was significantly lower than that in the normal group (Fig. 1). After 12 weeks of treatment, the 20 and 40 ng/kg doses of ELD significantly prevented the reduction of the areal BMD of the L2–L4 vertebrae and the proximal and distal femur that was observed in the vehicle group. ELD did not alter the areal BMD of the middle femur. ALF had no significant effect on the areal BMD of the L2–L4 vertebrae or the femur, other than that the 25 ng/kg dose of ALF significantly prevented the decrease of BMD in the distal femur (Fig. 1). In the L5 vertebra, the total and cortical volumetric BMD determined by pQCT was significantly lower in the vehicle group than in the normal group, and in the femoral diaphysis, the cortical volumetric BMD in the vehicle group was also lower than in the normal group (Table 2). The trabecular volumetric BMD in the vehicle group tended to be lower than that in the normal group, but not significantly. The 20 and 40 ng/kg doses of ELD significantly prevented the decreases in total and trabecular volumetric BMD in the L5 vertebra. ELD did not alter the cortical volumetric BMD in either the L5 vertebra or the femoral diaphysis. ALF did not have a significant effect on the volumetric BMD in the L5 vertebra. The 25 ng/kg dose of ALF significantly prevented the decrease in the cortical volumetric BMD in the femoral diaphysis (Table 2).

**Table 2:** Volumetric BMD of the L5 vertebra and femoral diaphysis after 12 weeks of treatment

	Normal	STZ						
		Vehicle	ELD (ng/kg)			ALF (ng/kg)		
			10	20	40	25	50	100
L5								
Total BMD (mg/cm^3^)	738.3 ± 20.0	630.5 ± 26.1^a^	661.2 ± 16.2	714.0 ± 16.5^b^	731.4 ± 20.9^b^	676.3 ± 19.6	640.3 ± 14.0	659.9 ± 23.8
Trabecular BMD (mg/cm^3^)	366.0 ± 21.0	308.1 ± 19.9	325.0 ± 12.9	380.0 ± 15.5^b^	411.6 ± 17.9^b^	348.7 ± 19.6	304.6 ± 6.5	343.3 ± 17.8
Cortical BMD (mg/cm^3^)	1097.2 ± 8.7	1053.0 ± 12.8^a^	1066.5 ± 11.2	1079.2 ± 8.4	1072.5 ± 10.8	1073.4 ± 15.2	1072.0 ± 9.9	1064.2 ± 11.8
Femoral diaphysis								
Cortical BMD (mg/cm^3^)	1441.9 ± 3.7	1410.0 ± 4.1^a^	1418.5 ± 4.8	1420.5 ± 4.5	1413.2 ± 4.6	1427.9 ± 4.9^b^	1416.3 ± 3.7	1412.9 ± 4.5

Data are presented as mean ± SEM (*n* = 8–12)

*STZ* streptozotocin, *ELD* eldecalcitol, *ALF* alfacalcidol, *BMD* bone mineral density

^a^ *P* < 0.05 versus normal rats by unpaired *t* test

^b^ *P* < 0.05 versus vehicle-treated rats by Dunnett’s multiple comparison test

### Bone Turnover Markers

Serum OC in the vehicle group was lower than that in the normal group at 4 weeks into the experimental period, and this reduction was maintained through 12 weeks (Table 3). Treatment with ELD or ALF did not affect serum OC except for the 100 ng/kg dose of ALF, in which serum OC at 8 weeks was significantly higher than in the vehicle group. Urinary DPD/Cr in the vehicle group tended to be higher than that in the normal group, but not significantly (Table 3). With all doses of ELD and with the 100 ng/kg dose of ALF, urinary DPD/Cr was significantly lower than in the vehicle group at 4 and 8 weeks of treatment, and these effects were continued through 12 weeks of treatment except for the 10 ng/kg ELD-treated group (Table 3).

**Table 3:** Bone turnover markers after 4, 8, and 12 weeks of treatment

			STZ						
		Normal	Vehicle	ELD (ng/kg)			ALF (ng/kg)		
				10	20	40	25	50	100
Serum OC (ng/mL)	4 weeks	23.95 ± 1.69	9.40 ± 0.65^a^	8.69 ± 1.35	9.25 ± 2.33	8.68 ± 0.95	8.45 ± 0.88	10.21 ± 0.79	11.71 ± 1.70
	8 weeks	16.38 ± 1.59	6.65 ± 0.43^a^	7.42 ± 1.48	6.78 ± 1.34	7.89 ± 0.81	10.11 ± 1.86	9.69 ± 0.59	10.51 ± 0.92^b^
	12 weeks	21.69 ± 1.63	7.70 ± 0.66^a^	9.86 ± 1.37	8.91 ± 1.83	9.61 ± 0.89	8.72 ± 1.24	9.74 ± 1.00	9.87 ± 1.02
Urinary DPD/Cr (nmol/mmol)	4 weeks	101.92 ± 11.01	130.67 ± 12.48	74.68 ± 8.91^b^	49.19 ± 7.65^b^	61.37 ± 12.47^b^	110.03 ± 13.74	109.11 ± 13.58	77.29 ± 12.03^b^
	8 weeks	52.11 ± 3.84	40.31 ± 9.76	40.31 ± 5.69^b^	35.26 ± 7.87^b^	30.73 ± 4.21^b^	56.13 ± 8.97	58.90 ± 7.50	41.62 ± 4.67^b^
	12 weeks	41.19 ± 2.75	44.51 ± 5.57	36.08 ± 4.10	27.84 ± 2.91^b^	25.06 ± 3.21^b^	40.76 ± 3.72	48.76 ± 5.06	24.31 ± 2.13^b^

Data are presented as mean ± SEM (*n* = 9–12)

*STZ* streptozotocin, *ELD* eldecalcitol, *ALF* alfacalcidol, *OC* osteocalcin, *DPD* deoxypyridinoline, *Cr* creatinine

^a^ *P* < 0.05 versus normal rats by unpaired *t* test

^b^ *P* < 0.05 versus vehicle-treated rats by Dunnett’s multiple comparison test

### Bone Histomorphometry

STZ-induced DM rats showed a significant reduction in BV/TV of the L3 vertebra, accompanied by a significant decrease in Tb.Th and a significant increase in Tb.Sp compared to the normal rats (Table 4). All doses of ELD resulted in significant increases in BV/TV relative to the vehicle group. The increase in BV/TV observed in the ELD-treated groups was accompanied by a significant increase in Tb.N as well as Tb.Th and a significant decrease in Tb.Sp. The highest dose of ALF was associated with a significant increase in BV/TV as compared to the vehicle group accompanied by a significant increase in Tb.N and a significant decrease in Tb.Sp (Table 4).

**Table 4:** Structural parameters in trabecular bone of the L3 vertebra and cortical bone of the femoral diaphysis after 12 weeks of treatment

	Normal	STZ						
		Vehicle	ELD (ng/kg)			ALF (ng/kg)		
			10	20	40	25	50	100
L3								
BV/TV (%)	26.9 ± 2.0	16.6 ± 1.0^a^	24.9 ± 1.0^b^	24.4 ± 1.2^b^	26.1 ± 1.6^b^	19.1 ± 1.6	20.2 ± 1.5	22.0 ± 1.0^b^
Tb.Th (μm)	85.7 ± 4.4	56.5 ± 2.7^a^	72.3 ± 2.2^b^	66.8 ± 2.6^b^	75.2 ± 4.0^b^	61.6 ± 3.7	65.5 ± 4.3	64.7 ± 2.6
Tb.N (/mm)	3.15 ± 0.17	2.92 ± 0.09	3.44 ± 0.10^b^	3.65 ± 0.11^b^	3.46 ± 0.08^b^	3.08 ± 0.15	3.07 ± 0.12	3.42 ± 0.09^b^
Tb.Sp (μm)	240.8 ± 20.9	289.2 ± 10.7^a^	221.1 ± 9.4^b^	209.5 ± 8.8^b^	215.7 ± 9.1^b^	272.0 ± 20.9	264.8 ± 13.0	230.5 ± 8.3^b^
Femur								
T.Ar (mm^2^)	14.0 ± 0.5	11.9 ± 0.4^a^	12.0 ± 0.2	11.3 ± 0.2	12.3 ± 0.4	12.0 ± 0.3	12.3 ± 0.3	12.1 ± 0.2
Ma.Ar (mm^2^)	4.54 ± 0.24	4.73 ± 0.19	4.56 ± 0.15	4.02 ± 0.22^b^	4.43 ± 0.20	4.24 ± 0.12	4.57 ± 0.15	4.56 ± 0.16
Ct.Ar (mm^2^)	9.49 ± 0.29	7.21 ± 0.27^a^	7.49 ± 0.19	7.26 ± 0.12	7.84 ± 0.25	7.75 ± 0.26	7.68 ± 0.24	7.50 ± 0.18
Ct.Wi (mm)	0.860 ± 0.018	0.667 ± 0.022^a^	0.705 ± 0.018	0.721 ± 0.016	0.732 ± 0.019	0.738 ± 0.020^b^	0.718 ± 0.018	0.707 ± 0.018

Data are presented as mean ± SEM (*n* = 9–12)

*STZ* streptozotocin, *ELD* eldecalcitol, *ALF* alfacalcidol

^a^ *P* < 0.05 versus normal rats by unpaired *t* test

^b^ *P* < 0.05 versus vehicle-treated rats by Dunnett’s multiple comparison test

In the cortical bone in the femoral diaphysis, STZ-induced DM rats had a significant decrease in T.Ar, Ct.Ar, and Ct.Wi compared to the normal rats (Table 4). ELD did not change these parameters. However, the 20 ng/kg dose of ELD significantly lowered Ma.Ar compared to the vehicle group. ALF did not affect the structural parameters of the cortical bone except that the 25 ng/kg dose of ALF significantly increased Ct.Wi compared to the vehicle group (Table 4).

DM rats showed significant increases in bone resorption parameters Oc.S/BS and ES/BS, and significant decreases in bone formation parameters Ob.S/BS, OS/BS, MS/BS, MAR, and BFR/BS compared to the normal group (Table 5). ELD and ALF significantly reduced the DM-associated increases in bone resorption parameters, but did not alter bone formation parameters (Table 5).

**Table 5:** Dynamic parameters in trabecular bone of the L3 vertebra after 12 weeks of treatment

	Normal	STZ						
		Vehicle	ELD (ng/kg)			ALF (ng/kg)		
			10	20	40	25	50	100
Oc.S/BS (%)	3.14 ± 0.62	8.29 ± 0.54^a^	3.76 ± 0.41^b^	2.05 ± 0.47^b^	0.74 ± 0.16^b^	3.39 ± 0.58^b^	2.16 ± 0.27^b^	0.95 ± 0.18^b^
ES/BS (%)	12.26 ± 1.48	28.44 ± 1.66^a^	16.75 ± 1.56^b^	8.56 ± 1.68^b^	3.43 ± 0.61^b^	12.32 ± 1.72^b^	11.03 ± 0.82^b^	4.51 ± 0.78^b^
Ob.S/BS (%)	6.87 ± 0.60	2.32 ± 0.59^a^	1.58 ± 0.54	0.86 ± 0.52	1.94 ± 0.55	1.66 ± 0.47	3.01 ± 0.67	1.79 ± 0.47
OS/BS (%)	12.45 ± 0.72	3.31 ± 0.80^a^	2.32 ± 0.76	1.22 ± 0.70	3.54 ± 0.90	2.60 ± 0.69	4.54 ± 0.96	3.10 ± 0.77
MS/BS (%)	16.70 ± 1.57	5.33 ± 1.30^a^	2.56 ± 1.01	1.74 ± 1.16	3.03 ± 0.78	3.49 ± 1.20	6.55 ± 1.57	2.99 ± 0.77
MAR (μm/day)	1.38 ± 0.04	0.82 ± 0.06^a^	0.82 ± 0.13	0.44 ± 0.15	0.66 ± 0.12	0.87 ± 0.13	1.15 ± 0.12	0.85 ± 0.14
BFR/BS (mm^3^/mm^2^/year)	0.084 ± 0.008	0.018 ± 0.005^a^	0.011 ± 0.005	0.007 ± 0.005	0.010 ± 0.003	0.015 ± 0.008	0.031 ± 0.008	0.012 ± 0.003

Data are presented as mean ± SEM (*n* = 9–12)

*STZ* streptozotocin, *ELD* eldecalcitol, *ALF* alfacalcidol

^a^ *P* < 0.05 versus normal rats by unpaired *t* test

^b^ *P* < 0.05 versus vehicle-treated rats by Dunnett’s multiple comparison test

In DM rats, MI.BV/BV and MI.BS/BS were lower than in the normal group (Table 6). The highest dose of ELD and the 50 ng/kg dose of ALF significantly increased bone minimodeling parameters N.MI/BS, MI.BV/BV, and MI.BS/BS compared to the vehicle group. The 100 ng/kg dose of ALF also significantly increased MI.BV/BV (Table 6).

**Table 6:** Bone minimodeling in trabecular bone of the L3 vertebra after 12 weeks of treatment

	Normal	STZ						
		Vehicle	ELD (ng/kg)			ALF (ng/kg)		
			10	20	40	25	50	100
N.MI/BS (/mm)	0.184 ± 0.021	0.131 ± 0.028	0.190 ± 0.062	0.161 ± 0.062	0.464 ± 0.100^b^	0.373 ± 0.069	0.501 ± 0.089^b^	0.347 ± 0.100
MI.BV/BV (%)	0.326 ± 0.056	0.172 ± 0.037^a^	0.438 ± 0.116	0.546 ± 0.216	1.302 ± 0.405^b^	0.935 ± 0.213	1.561 ± 0.318^b^	1.166 ± 0.340^b^
MI.BS/BS (%)	1.578 ± 0.201	0.887 ± 0.199^a^	1.830 ± 0.489	1.811 ± 0.786	4.793 ± 1.183^b^	3.291 ± 0.653	5.492 ± 1.139^b^	3.616 ± 1.002

Data are presented as mean ± SEM (*n* = 9-12)

*STZ* streptozotocin, *ELD* eldecalcitol, *ALF* alfacalcidol, *N.MI* number of minimodeling, *MI.BV* minimodeling bone volume, *MI.BS* minimodeling bone surface

^a^ *P* < 0.05 versus normal rats by unpaired *t* test

^b^ *P* < 0.05 versus vehicle-treated rats by Dunnett’s multiple comparison test

### Bone Strength

STZ-induced DM rats showed significant decreases in maximum load and stiffness in the L5 vertebral body compared to the normal group (Table 7). Treatment with 40 ng/kg of ELD significantly prevented DM-associated reduction in maximum load, but ALF did not affect biomechanical strength of the L5 vertebral body (Table 7).

**Table 7:** Biomechanical properties of the L5 vertebral body and femoral shaft and AGE content after 12 weeks of treatment

	Normal	STZ						
		Vehicle	ELD (ng/kg)			ALF (ng/kg)		
			10	20	40	25	50	100
L5								
Maximum load (N)	376 ± 32	225 ± 20^a^	235 ± 14	270 ± 17	298 ± 23^b^	282 ± 22	260 ± 22	255 ± 19
Stiffness (N/mm)	1661.0 ± 161.2	1203.6 ± 138.2^a^	1148.5 ± 82.4	1291.6 ± 111.8	1363.9 ± 104.0	1587.7 ± 120.1	1394.2 ± 131.8	1467.0 ± 90.1
Work to failure (mJ)	63.8 ± 8.2	40.8 ± 7.2	40.0 ± 7.5	48.9 ± 7.3	46.6 ± 5.6	36.0 ± 3.8	34.1 ± 3.7	38.2 ± 6.9
Femur								
Maximum load (N)	285 ± 9	206 ± 9^a^	208 ± 8	204 ± 13	230 ± 7	229 ± 9	220 ± 12	201 ± 11
Stiffness (N/mm)	633.7 ± 31.4	494.1 ± 22.7^a^	538.0 ± 22.7	537.5 ± 19.9	524.6 ± 11.7	545.4 ± 14.3	544.6 ± 15.9	501.9 ± 15.8
Work to failure (mJ)	152.2 ± 9.7	95.2 ± 4.2^a^	106.1 ± 7.5	86.2 ± 7.1	109.2 ± 3.5	107.5 ± 5.8	105.8 ± 9.8	89.0 ± 8.4
Ultimate stress (MPa)	124.0 ± 3.3	119.7 ± 4.6	123.8 ± 4.3	123.7 ± 8.8	127.6 ± 4.0	127.2 ± 3.1	118.1 ± 3.8	115.6 ± 5.2
Young’s modulus (MPa)	1866.2 ± 74.7	2157.1 ± 118.2	2331.1 ± 102.4	2433.2 ± 130.8	2114.9 ± 97.0	2205.7 ± 96.3	2136.4 ± 59.5	2113.4 ± 92.1
Toughness (MPa)	9.76 ± 0.58	7.41 ± 0.27^a^	8.77 ± 0.63	6.98 ± 0.57	8.41 ± 0.27	8.35 ± 0.43	7.85 ± 0.64	7.09 ± 0.64
AGEs (ng quinine/mg of collagen)	46.45 ± 3.34	63.11 ± 2.64^a^	60.09 ± 2.12	64.86 ± 2.96	68.91 ± 4.57	66.10 ± 3.13	63.41 ± 2.71	68.59 ± 2.84

Data are presented as mean ± SEM (*n* = 8–12)

*STZ* streptozotocin, *ELD* eldecalcitol, *ALF* alfacalcidol, *AGEs* advanced glycation end products

^a^ *P* < 0.05 versus normal rats by unpaired *t* test

^b^ *P* < 0.05 versus vehicle-treated rats by Dunnett’s multiple comparison test

In the 3-point bending test of the femoral shaft, DM rats had significant decreases in maximum load, stiffness, work to failure, and toughness compared to the normal group (Table 7). ELD and ALF did not change any biomechanical strength parameters of the femoral shaft (Table 7).

### Collagen Crosslinks

AGE content in the femoral cortical bone was significantly greater in the vehicle group than in the normal group. ELD and ALF did not affect AGE content (Table 7).

## Discussion

In this study, a reduction in bone biomechanical strength with decreases in BMD and bone quality was observed in STZ-induced type I DM rats. Treatment with ELD showed a protective effect on biomechanical strength with prevention of trabecular bone loss.

A reduction in BMD is a risk factor for fragility fractures; however, reduction in bone quality, including deterioration of bone microstructure and collagen crosslinks, is also a critical factor in bone fragility [28, 29]. Silva et al. reported the reduction of bone biomechanical strength in STZ-induced DM rats, which was associated with decreased BMD, geometric changes in bone microarchitecture, and accumulation of non-enzymatic collagen crosslinks in bone [30]. In the present study, a reduction in biomechanical strength of the lumbar vertebrae and femoral shaft was observed in STZ-induced DM rats. This was consistent with the decreases in areal and volumetric BMD, the deterioration of bone microstructure, and the increase in AGE content in bone. These observations are in line with the findings from previous reports [30, 31].

In our STZ-induced DM rats, urinary DPD concentration showed a tendency to increase, and bone resorption parameters (Oc.S/BS and ES/BS) in histomorphometric analysis significantly increased compared to normal rats. These results suggested that bone resorption was enhanced in our DM model. On the other hand, bone formation declined in the STZ-induced DM rats, which was demonstrated as decreases in serum OC concentration and bone formation parameters (Ob.S/BS, OS/BS, MS/BS, MAR, and BFR/BS) in histomorphometric analysis. Although the results concerning the reduction in bone formation in STZ-induced DM animals are consistent, the results concerning changes in bone resorption are controversial [31–34]. Hie et al. reported an increase in osteoclastogenesis accompanied with an increase in RANK expression, and a reduction in osteoblastogenesis with a decrease in Runx 2 expression in STZ rats [33]. On the other hand, Hamada et al. observed a reduction in both bone formation and bone resorption in STZ mice by bone histomorphometry [32]. The status of bone resorption in STZ-induced DM might differ according to the species or the timing of observation.

Treatment with the highest dose of ELD prevented the DM-induced reduction in bone biomechanical strength while preventing BMD reduction and bone structural deterioration in the lumbar vertebrae. Analysis of bone turnover markers showed that ELD lowered bone resorption as evidenced by a decrease in urinary DPD excretion, but did not influence bone formation as shown by the absence of change in serum OC in DM rats. These observations were also confirmed with bone histomorphometric analysis, in which bone resorption parameters were decreased but bone formation parameters were not changed by ELD treatment.

Del Pino-Montes et al. reported that active vitamin D_3_ prevented bone loss in STZ-induced DM rats under a condition in which insulin was administered to maintain blood glucose below 350 mg/dL. This preventive effect on bone was induced by the improvement of DM via prevention of β cell damage in the pancreas by active vitamin D_3_ [35]. ELD did not change the blood glucose or HbA1c in our study; therefore, an indirect effect of ELD via improvement of DM was not included in the effects of ELD on the prevention of trabecular bone loss in this study.

DM is frequently accompanied by complications, such as neuropathy, which often causes motor disturbance [36]. Hao et al. showed that hyperglycemia promoted de-differentiation of Schwann cells into immature cells and that active vitamin D_3_, including ELD, reduced de-differentiation of Schwann cells via elevation of IGF-1 expression, resulting in the improvement of nerve function as well as motor function in DM mice [37]. Muscle and bone tissues are in a close relationship, and physical limitation is one of the risk factors of bone loss [38]. We did not examine the exercise capacity of DM rats in this study; therefore, it is unclear how motor function changes and how motor disturbance is related to bone loss in this study. However, we cannot exclude the possibility that ELD may prevent bone loss, in part, through the improvement of motor function.

It has been reported that the accumulation of non-enzymatic AGE crosslinks in bone is associated with bone fragility in DM [13, 30, 39, 40]. The accumulation of AGEs in femoral cortical bone in DM rats was confirmed in this study; however, ELD did not change it. Saito et al. reported that ELD administered to OVX monkeys increased enzymatic crosslinks and reduced AGE crosslinks in bone, and suggested that newly produced collagen matrix by ELD-induced bone minimodeling may contribute to the reduction of AGEs [20]. In our study, bone formation in STZ-induced DM rats was severely restricted and ELD did not alter it. Therefore, the contribution of minimodeling by ELD to the reduction of AGE accumulation in bone may be minimal in DM rats. Actually, minimodeling bone volume per total bone volume in the 40 ng/kg ELD-treated group was only 1.3% in this study, while about 10% of minimodeling bone volume was observed by ELD treatment in OVX rats in a previous study [19].

In this study, we compared the effects of ELD and ALF on bone metabolism in STZ-induced DM rats. ELD improved the bone biomechanical strength, BMD, and microstructure of the lumbar vertebrae in STZ rats. ALF, however, did not induce significant changes in biomechanical strength or BMD, and only at the highest dose of ALF was structural deterioration prevented. Serum Ca concentrations and levels of urinary Ca excretion in ELD-treated groups were similar to those in ALF-treated groups. Uchiyama et al. previously reported a comparison of the influence of ELD and ALF on bone metabolism in OVX rats; they showed that ELD had a greater activity than ALF in increasing BMD and reducing bone resorption when administered at doses that elicited the same potency in the effects on serum and urinary Ca [16]. This superiority of ELD over ALF on bone metabolism was confirmed even in STZ-induced DM rats.

In conclusion, treatment with ELD prevented the reduction of bone biomechanical strength with the improvement of BMD and bone microstructure in trabecular bone in STZ-induced type I DM rats. These results are expected to clarify the usefulness of ELD in patients with diabetic osteoporosis.
